# Development of a stable transgenic *Theileria equi* parasite expressing an enhanced green fluorescent protein/blasticidin S deaminase

**DOI:** 10.1038/s41598-021-88594-w

**Published:** 2021-04-27

**Authors:** Bumduuren Tuvshintulga, Arifin Budiman Nugraha, Tomoka Mizutani, Mingming Liu, Takahiro Ishizaki, Thillaiampalam Sivakumar, Xuenan Xuan, Naoaki Yokoyama, Ikuo Igarashi

**Affiliations:** 1 National Research Center for Protozoan Diseases, Obihiro University of Agriculture Veterinary Medicine, Inada-cho, Obihiro, Hokkaido 080-8555 Japan; 2 Institute of Veterinary Medicine, Mongolian University of Life Sciences, Zaisan, Ulaanbaatar, 17024 Mongolia; 3 Department of Animal Infectious Diseases and Veterinary Public Health, Faculty of Veterinary Medicine, IPB University, Jl. Agatis, Kampus IPB Dramaga, Bogor, Jawa Barat 16680 Indonesia; 4 Department of Protozoology, Institute of Tropical Medicine (NEKKEN), Nagasaki University, Nagasaki, 852-8523 Japan

**Keywords:** Parasitology, Parasite biology

## Abstract

*Theileria equi*, an intraerythrocytic protozoan parasite, causes equine piroplasmosis, a disease which negatively impacts the global horse industry. Genetic manipulation is one of the research tools under development as a control method for protozoan parasites, but this technique needs to be established for *T*. *equi*. Herein, we report on the first development of a stable transgenic *T*. *equi* line expressing enhanced green fluorescent protein/blasticidin S deaminase (eGFP/BSD). To express the exogenous fusion gene in *T*. *equi*, regulatory regions of the *elongation factor-1 alpha* (*ef-1α*) gene were identified in *T. equi*. An eGFP/BSD-expression cassette containing the *ef-1α* gene promoter and terminator regions was constructed and integrated into the *T*. *equi* genome. On day 9 post-transfection, blasticidin-resistant *T*. *equi* emerged. In the clonal line of *T. equi* obtained by limiting dilution, integration of the eGFP/BSD-expression cassette was confirmed in the designated B*-*locus of the *ef-1α* gene via PCR and Southern blot analyses*.* Parasitaemia dynamics between the transgenic and parental *T*. *equi* lines were comparable in vitro. The eGFP/BSD-expressing transgenic *T*. *equi* and the methodology used to generate it offer new opportunities for better understanding of *T*. *equi* biology, with the add-on possibility of discovering effective control methods against equine piroplasmosis.

## Introduction

Equine piroplasmosis is a tick-borne infectious disease of horses caused by *Theileria equi* or *Babesia caballi*. Both parasites are intraerythrocytic haemoprotozoans within the order Piroplasmida (phylum Apicomplexa)^1^. Equine piroplasmosis often causes huge economic losses to the equine industry, particularly by impairing international trade and sport^2,3^. *T. equi*, which is more pathogenic than *B*. *caballi*, is widely distributed in tropical and subtropical regions, coinciding with the distribution of its tick vectors^4^. Horses with acute or sub-acute infection stages of *T*. *equi* may develop severe clinical signs, including fever, hemoglobinuria, anemia, and icterus^2^. Should infected horses recover from this clinical disease, they become carriers of *T*. *equi* infection for several years and the parasites persist in many of their organs, including the bone marrow, liver, spleen, lungs, heart, and brain^5,6^. Vaccine unavailability, coupled with the low efficacy and toxic-side effects of currently used drugs, emergence of drug-resistant parasites, and the rapid development of acaricide resistance in ticks make controlling *T. equi* infection extremely difficult^7–9^. Hence, although development of improved control measures is vital, it requires a comprehensive understanding of the biology of *T*. *equi*.

In common with other *Theileria* species, *T*. *equi* sporozoites, when injected into the host by infected ticks, invade leukocytes where they undergo schizogony to form schizonts in the infected cells^10–12^. When schizonts rupture they release merozoites, which after infecting other red blood cells (RBCs) undergo merogony (asexual reproduction) and produce more merozoites^2^. These merozoites infect uninfected RBCs, where they continue to proliferate during merogony. Detailed information about invasion, schizont formation, and asexual reproduction is, however, scant for *T*. *equi*, and this undermines the development of control and preventive strategies against this parasitic disease. The tick vectors that are competent to be parasitised by *T*. *equi* parasites acquire them when feeding on infected animals, and these parasites undergo sexual reproduction in the tick midgut^13^. As with the asexual stages in the equine host, however, the sexual stages of *T*. *equi*—which occur in the tick vector—require thorough investigation. Similar to other *Theileria* species, *T*. *equi* persists transstadially in the subsequent tick stages, which then transmit the infection to other non-infected equines^2,12,14^. However, transovarial transmission in ticks, which is a unique feature of *Babesia*, cannot be entirely ruled out for *T*. *equi*^14^. Therefore, research to clarify the developmental stages of *T*. *equi* in horses and ticks will be essential for achieving effective control of this economically significant parasite species. The lack of advanced research tools is certainly one of the major constraints in *T*. *equi* research.

Genetic manipulation helps researchers to perform experiments wherein the gain or loss of specific gene expression and function is achieved in a pathogen. This can provide researchers with fresh insights into a parasite’s life cycle, pathogenesis and host–parasite interactions, for example. Recently, enhanced green fluorescent protein (eGFP) has been widely used as a reporter for the transfection of apicomplexan parasites, such as *T*. *parva*, *T*. *annulata*, *B. bovis*, *B. bigemina*, *B. ovata*, *B. gibsoni*, *Plasmodium falciparum*, *Toxoplasma gondii*, and *Cryptosporidium parvum*^15–25^. More recently, a fusion gene encoding eGFP/blasticidin S deaminase (eGFP/BSD) was successfully introduced as a reporter and drug-selectable marker for developing stable transgenic *B. bovis* and *B. bigemina* lines^17,18,24,25^. Genetic manipulations using these parasites allowed researchers to clarify the mechanisms underlying cellular invasion, identify vaccine and drug candidates, and conduct cutting-edge research to develop effective preventive and control measures^24–26^.

Despite these advances in apicomplexan research, tools for genetic manipulation of *T*. *equi* have not been developed. Hence, we aimed to establish a method for stable transformation of *T*. *equi* by integrating the eGFP/BSD gene into this parasite’s genome.

## Results

### Inhibitory effect of blasticidin on a parental strain of *T. equi* in vitro

To determine the appropriate blasticidin concentration to use as the selection marker for the eGFP/BSD transgenic parasites, the inhibitory effect of blasticidin was tested on the in vitro growth of the *T*. *equi* USDA strain. Blasticidin was found to significantly inhibit the growth of the parental line in a dose-dependent manner, and its half maximal inhibitory concentration (IC_50_) was determined to be 7.0 ± 0.91 µM (Supplementary Fig. S1).

### Construction of a transfer plasmid carrying the eGFP/BSD-expression cassette for use in *T. equi*

**Figure 1:**
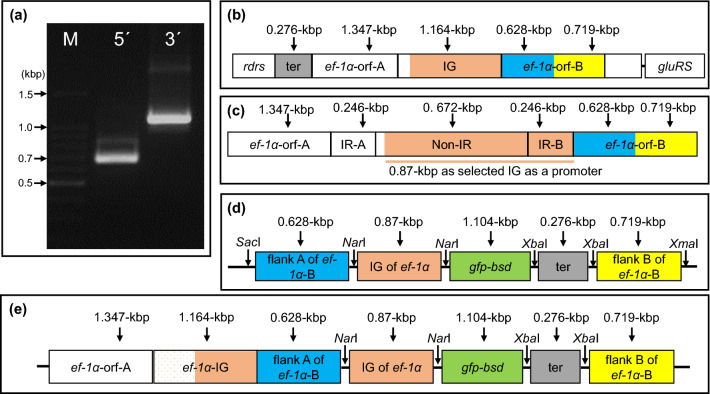
Construction of the enhanced green fluorescent protein/blasticidin S deaminase (eGFP/BSD)-expression cassette targeting the *elongation factor-1α-B* gene locus in *T*. *equi* genome. (**a**) Visualisation of the amplicons obtained from 5′-RACE PCR (5′) and 3′-RACE PCR (3′). M, 100 base-pair DNA ladder marker. (**b**) Structure of head-to-head orientation of two copies of *elongation factor-1α* (*ef-1α*) genes in the *T*. *equi* (USDA strain) genome. The *ef-1α* gene loci are flanked by *ribonucleoside-diphosphate reductase* (*rdrs*) and *glutamyl-tRNA synthetase* (*gluRS*) genes. Regions containing the terminator (ter), a part of the intergenic region (IG), homologous flanking fragment A (flank A), and homologous flanking fragment B (flank B) that were selected for plasmid construction are highlighted in grey, orange, blue, and yellow, respectively. (**c**) Magnified structure of the IG containing inverted region-A (IR-A), non-inverted region, and inverted region-B (IR-B). (**d**) The eGFP/BSD-expression cassette. The expression cassette included *green fluorescent protein* (*gfp*) and *blasticidin S deaminase* (*bsd*) genes. (**e**) Genomic integration of the eGFP/BSD-expression cassette into the *ef-1α-B* gene locus of *T*. *equi*.

The *ef-1α* gene’s promoter was used to express the eGFP/BSD gene in *T. equi.* To identify the promoter region in the *T*. *equi* USDA strain’s genome, the mRNA sequence of the *ef-1α* gene was determined using the rapid amplification of cDNA ends (RACE) technique. Briefly, two amplicons of approximately ~ 0.7- and ~ 1.1-kilo base pairs (kbp) long were obtained from the 5′- and 3′-RACE PCR reactions, respectively (Fig. 1a). These two amplicons were cloned and sequenced. Based on the resultant mRNA sequences from the USDA strain, we identified two intron-lacking loci belonging to the *ef-1α* gene (1.347-kbp); these two sequences were also found on chromosome 2 of the *T*. *equi* WA strain (GenBank accession no. NW_004668230.1; Gene ID no. BEWA_037990 (695,088–696,737) and BEWA_038000 (697,369–698,964), respectively). The two loci in the *T*. *equi* WA strain contain two *ef-1α* head-to-head-oriented ORFs flanked by *ribonucleoside-diphosphate reductase* (*rdrs*; Gene ID: BEWA_037980) and *glutamyl*-*tRNA synthetase* (*gluRS*; Gene ID: BEWA_038010) genes. Therefore, we used a pair of forward and reverse primers targeting the *rdrs* and *gluRS* genes, respectively, to amplify a fragment of ~ 5.46-kbp from the genomic DNA (gDNA) of the *T*. *equi* USDA strain. The amplicon was cloned and sequenced (Fig. 1b). Our analysis confirmed that the amplified DNA fragment contained *ef-1α-A* gene, 1.164-kbp intergenic region (IG) containing 0.246-kbp inverted repeats at its both ends, and *ef-1α-B* gene (Fig. 1c). Based on the sequence, the promoter (0.87-kbp of the 1.164-kbp IG located closer to the *ef-1α-B* gene), terminator (0.278-kbp of the region located between *rdrs* and *ef-1α-A* genes) and two homologues flanking segments (0.628- and 0.719-kbp of the regions located at the 5′ and 3′ ends of the *ef-1α-B* gene, respectively) were PCR-amplified and then inserted into a Bluescript plasmid (Fig. 1d). In addition, to express the reporter gene and drug selectable marker in *T*. *equi*, a 1.104-kbp eGFP/BSD fusion gene amplified from the pTracer-CMV/Bsd Mammalian Expression Vector was inserted between the promoter and terminator segments in the constructed Bluescript plasmid described above. The nucleotide sequence of the eGFP/BSD-expression cassette for *T*. *equi* is deposited in GenBank (Accession No. MW021139). The eGFP/BSD-expression cassette was then targeted to the *ef-1α-B* gene locus in the genome of *T*. *equi* (Fig. 1e).

### Transfection and isolation of a transgenic eGFP/BSD-expressing *T. equi* line

**Figure 2:**
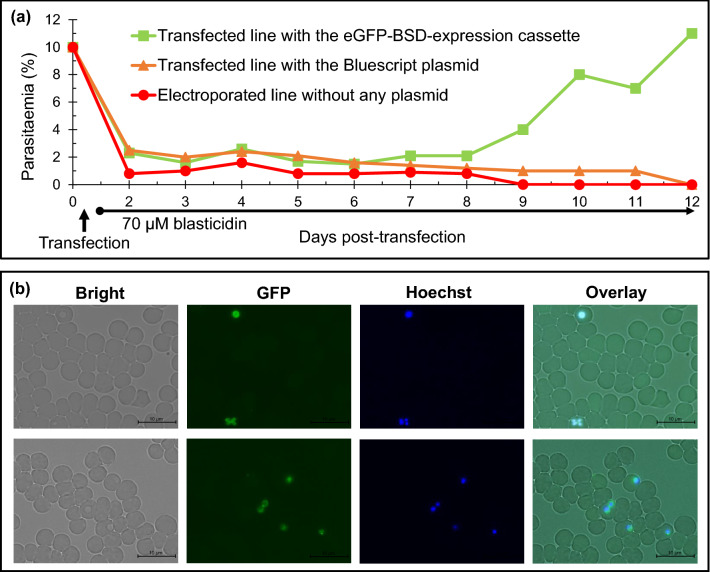
Transfection and isolation of the enhanced green fluorescent protein/blasticidin S deaminase (eGFP/BSD)-expressing transgenic *T*. *equi* parasite*.* (**a**) Selecting the *T*. *equi* transgenic line using blasticidin pressure. Blasticidin treatment was initiated on day 1 post-transfection. Blasticidin-resistant *T*. *equi* emerged on day 9 post-transfection. (**b**) Visualisation of *T*. *equi* at one year after the isolation of clonal line. A green fluorescent signal was emitted in both the merozoite (ring, paired, and Maltese cross forms) and trophozoite (ring form) stages of the transfected parasites.

**Figure 3:**
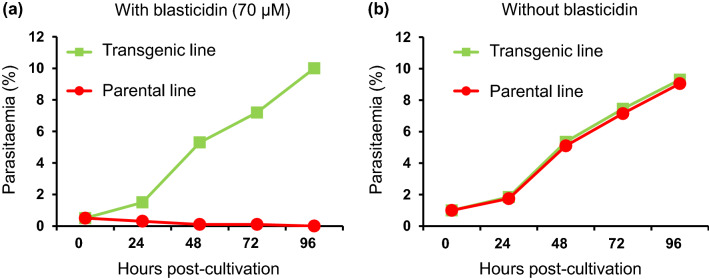
Parasitaemia curves of the transgenic *T*. *equi* line expressing enhanced green fluorescent protein/blasticidin S deaminase (eGFP/BSD) and the parental line. (**a**) Transgenic *T*. *equi* was resistant to blasticidin but the parental line was not when cultured in medium containing 70-µM blasticidin. (**b**) The parasitaemia dynamics were comparable between the transgenic and parent lines when cultured in normal medium.

*T*. *equi* was transfected with the linearised eGFP/BSD-expression cassette and then treated with 70-μM blasticidin (10 × IC_50_). Blasticidin-resistant parasites emerged 9 days after transfection (Fig. 2a). On day 12 post-transfection, a green fluorescent signal emission was observed in the parasite’s body (Supplementary Fig. S2). Subsequently, a clonal line of transgenic *T. equi* was isolated from the initial transfected parasites, using a limiting dilution method conducted under 70-μM blasticidin drug pressure. Even after the blasticidin pressure was discontinued, this green fluorescent signal could be observed in the transgenic clone for at least one year (Fig. 2b). When the transgenic and parental lines were treated with 70-μM blasticidin, the initial parasitaemia (~ 1%) in the transgenic line reached up to 11% within 96 h of treatment, whereas the parasitaemia decreased to below 0.1% in the parental line (Fig. 3a). The parasitaemia curve of the transgenic line was comparable to that of the parental *T. equi* line when both lines were cultured without blasticidin (Fig. 3b).

### Confirmation of genomic integration of the eGFP/BSD-expression cassette in transgenic *T. equi*

**Figure 4:**
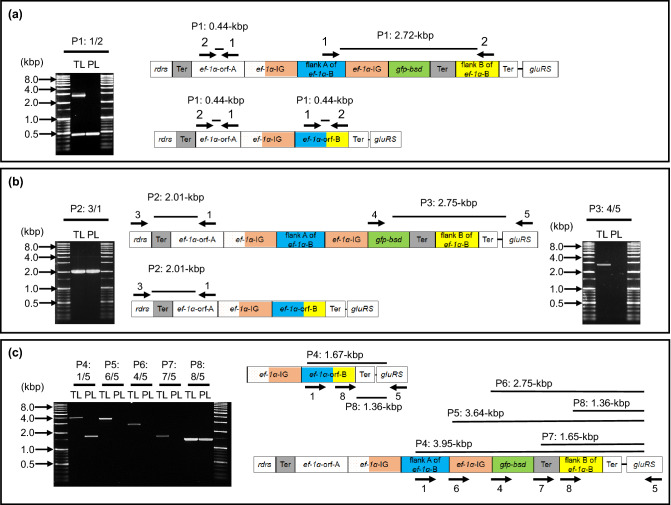
Graphical illustration of genomic integration of the enhanced green fluorescent protein/blasticidin S deaminase (eGFP/BSD)-expression cassette in transgenic *T*. *equi*. Genomic DNA extracted from the transgenic *T*. *equi* line (TL) and parental line (PL) was subjected to PCR assays. (**a**) A PCR assay (P1) confirmed the integration of the eGFP/BSD-expression cassette into one of the two *elongation factor-1α* (*ef-1α*) gene loci. (**b**) Two PCR assays (P2 and P3) confirmed that integration of the eGFP/BSD-expression cassette had occurred in the *ef-1α-B* gene locus. (**c**) Five PCR assays (P4–P8) confirmed the integration and correct orientation of the eGFP/BSD-expression cassette components in the *ef-1α-B* gene locus of the transgenic *T*. *equi*. A 1000 base-pair DNA ladder marker was used in all panels.

To confirm that genomic integration of the eGFP/BSD-expression cassette (2.264-kbp: *ef-1α* gene promoter, coding region of the eGFP/BSD gene and *ef-1α* gene terminator) had occurred in the transgenic line, PCR and Southern blot analyses were conducted to detect specific amplicons or length polymorphisms in the genome (Supplementary Table S1). When a set of forward and reverse primers targeting the homologous flanking A and B fragments was used for PCR, 0.446- and 2.722-kbp amplicons were detected as arising from the gDNA of the transgenic line, indicating that the eGFP/BSD-expression cassette had integrated only into one of the two loci of the *ef-1α* genes (Fig. 4a). By contrast, the same PCR primers only amplified the 0.446-kbp fragment from the gDNA of the parental line. Furthermore, when primers targeting *rdrs* and the homologous flanking fragment A of the *ef-1α* genes were used in a PCR, a 2.010-kbp fragment was amplified from the gDNA of both transgenic and parental lines (Fig. 4b, *left*), indicating that eGFP/BSD-expression cassette has not been integrated into the *ef-1α-A* gene locus which is located close to the *rdrs* gene. Conversely, when a primer pair targeting the eGFP/BSD and *gluRS* genes was used in PCR, a 2.759-kbp fragment was amplified from the gDNA of the transgenic line but not from the parental line (Fig. 4b, *right*). These results confirm that in the transgenic line, the eGFP/BSD-expression cassette had only integrated into the *ef-1α-B* gene locus located close to the *gluRS* gene. Moreover, our PCR assays conducted with gDNA from the transgenic line using forward primers that target flanking region A, the promoter, the eGFP/BSD coding region, the terminator, and flanking region B, together with the reverse primer targeting the *gluRS* gene, resulted in 3.952-, 3.642-, 2.759-, 1.652-, and 1.368-kbp amplicons, respectively (Fig. 4c). In contrast, when the gDNA of the parental line was used, PCRs targeting flanking region A and flanking region B of the *ef-1α* and *gluRS* genes amplified 1.676- and 1.368-kbp fragments, respectively, while no amplicon was detected in the remaining three PCRs. Overall, these PCR results further confirm the correct orientation of the eGFP/BSD-expression cassette and its integration into the *ef-1α-B* gene locus of the transgenic line.

**Figure 5:**
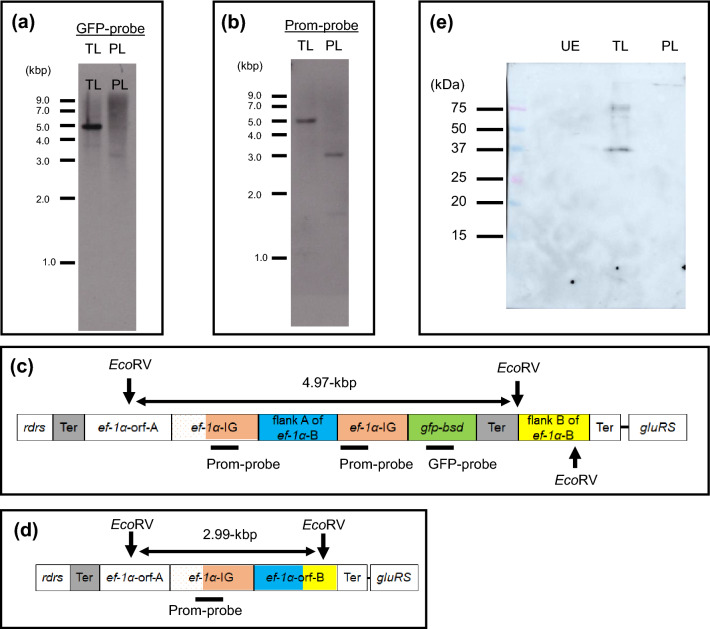
Graphical illustration of Southern blot and Western blot analyses. Genomic DNA extracted from the transgenic *T*. *equi* line (TL) and parental line (PL) was subjected to Southern blotting analyses after digestion with *Eco*RV. (**a**) A probe targeting the green fluorescent protein gene (GFP-probe) detected a single DNA fragment (4.9-kbp) in the genomic DNA (gDNA) obtained from the transgenic TL, but no such fragment was detectable in the PL. (**b**) A probe targeting the promoter region of the *ef-1α* gene (Prom-probe) detected a single 4.97-kbp fragment in the gDNA from the transgenic TL, whereas a 2.99-kbp fragment was detected in the PL. (**c**) Graphical illustration of *ef-1α* gene loci in the genome of the transgenic TL and *Eco*RV recognition sites. (**d**) Graphical illustration of *ef-1α* gene loci in the PL genome and *Eco*RV recognition sites. (**e**) In Western blot, an anti-GFP rabbit polyclonal antibody recognized a 40-kilodalton protein, corresponding to the size of eGFP/BSD, in lysate of TL but not in the lysates of PL and uninfected erythrocytes (UE).

We prepared two types of DNA probes for Southern blot analyses based on the eGFP/BSD gene and promotor region, which detected a 4.976-kbp fragment in the *Eco*RV restriction enzyme-digested gDNA of the transgenic line (Fig. 5a–c, and Supplementary Fig. S3 and S4). This indicates that the eGFP/BSD-expression cassette had only integrated into a single genomic locus. By contrast, in the Southern blotting experiment that used similarly digested gDNA from the parental *T. equi* line, a single 2.994-kbp fragment was detected by the promotor-targeting probe, but no band was detected by the probe targeting the eGFP/BSD gene (Fig. 5a,b,d, and Supplementary Fig. S3 and S4). In Western blot performed with a lysate from transgenic *T. equi*, an anti-GFP rabbit polyclonal antibody recognized a 40-kilodalton protein, corresponding to the size of eGFP/BSD, whereas no protein was detected when lysates from parental line and uninfected equine erythrocytes were analysed (Fig. 5e). Overall, our findings indicate that the eGFP/BSD-expression cassette successfully integrated only into the *ef-1α-B* gene locus in *T*. *equi,* and that the newly generated transgenic line stably expresses eGFP/BSD.

## Discussion

Successful cell transformation necessitates a cell-specific element that can bind to essential transcription factors and function as a strong promoter to drive exogenous genes into the target cells^27^. A suitable-for-transformation promoter region has not been identified in *T*. *equi*. Previous studies have reported that an intergenic region (IG) in the *ef-1α* gene contains strong promotor activity in piroplasma parasites such as *T*. *parva*, *B*. *bovis*, *B*. *bigemina*, *B*. *ovata* and *B*. *gibsoni*^15,19,28–30^. Therefore, we opted to use the *ef-1α* gene’s promoter, which successfully drove expression of the exogenous eGFP/BSD gene in *T*. *equi*. The *ef-1α* is a multi-copy gene in most eukaryotic cells; it encodes a regulatory protein, named as EF-1α, which is involved in translocating aminoacyl-tRNA to the ribosome^31^. Two *ef-1α* gene copies exist in the genomes of *Plasmodium* species and several *Babesia* species, including *B*. *bovis*, *B*. *bigemina*, *B*. *ovata* and *B*. *gibsoni*^19,20,28,29,32^. Previous studies have shown that the IG region functions as a bidirectional promoter and drives the expressions of the two *ef-1α* genes in *B*. *bovis*, *B*. *bigemina* and *B*. *ovata*^19,28,29^. In the present study, a similar bidirectional head-to-head-orientation of the two *ef-1α* gene copies was identified in the *T*. *equi* genome. In contrast, only a single copy of the *ef-1α* gene was reported in *T*. *annulata*, *T*. *parva* and *T*. *orientalis* (Gene IDs: TA06720, TP01_0726, TOT_010000685, respectively; https://piroplasmadb.org/piro/)^33,34^. Thus, generating transgenic parasites by integrating exogenous genes into the *ef-1α* gene locus is impossible in the aforementioned bovine *Theileria* parasites.

In *B*. *bigemina*, knocking out one of the two *ef-1α* gene copies resulted in a 3-times higher parasitaemia level in the transgenic line in comparison with that of the parental line, whereas the parasitaemia levels of transgenic *B*. *bovis* and its parental line were indistinguishable^18^. Similar to that found in *B*. *bovis*, knocking out the *ef-1α-B* gene in *T*. *equi* did not alter the parasitaemia level, when compared with that of the parental line. These findings suggest that our newly-developed transgenic *T*. *equi* is similar to the parental line in that it can invade, replicate and egress.

Our microscopic observations on the transgenic *T*. *equi* revealed that eGFP/BSD was stably expressed in all of its intraerythrocytic stages. A previous study demonstrated that *ef-1α* gene promoter stably drives expression of exogenous genes in *P*. *falciparum* throughout its life cycle^21^. Therefore, the transgenic *T. equi* may also express eGFP/BSD in all parasite stages in the equine host and tick vectors. However, only the experimental infections with transgenic *T. equi* will confirm our assumption.

The transgenic *T*. *equi* line developed herein will enable better visualisation of live parasites, and this is useful because the developmental stages of *T. equi* have not currently been investigated thoroughly enough in host blood cells. The lack of schizonticidal drugs makes complete clearance of *T. equi* from infected horses very difficult, and research on drug development is severely constrained because the formation and persistence of *T*. *equi* schizonts in infected horses awaits proper elucidation. Current knowledge on RBC invasion and asexual reproduction in *T*. *equi* is also incomplete. Notably, the invasion process, which is aided by the gliding motility of *Babesia* merozoites, is largely unknown in *T*. *equi*. The transgenic *T*. *equi* newly generated in the present study offers a powerful research tool for investigating the developmental stages of the parasite in its host blood cells. Its use provides opportunities for gaining fresh insight into schizont formation, RBC invasion, and asexual reproduction.

Vaccination against the equine piroplasmosis caused by *T*. *equi* would be one useful control method for minimizing economic losses in the equine industry^1,2^. However, to date, no vaccines are available for equine piroplasmosis. Recently, several antigens have been evaluated as vaccine candidates against various haemoprotozoan diseases^1,35–37^. Generally, such candidates are usually screened in vitro for their neutralisation sensitivities using antisera produced against them^35^. However, this strategy provides indirect evidence and has sometimes proved unsuitable because the candidate antigens can elicit non-specific immunity^24^. In contrast, gene knockout can provide a definitive answer as to whether the antigens under investigation are indispensable for parasite survival. Therefore, the methodology that we have used to develop transgenic *T*. *equi* would greatly facilitate the identification of vaccine candidates against equine piroplasmosis. In addition to the potential application in vaccine development, this technique may also be useful for discovering novel drug targets in *T*. *equi*.

In summary, we have developed the first stable eGFP/BSD-expressing transgenic *T. equi* line. Our transgenic *T*. *equi* and the methodology used to generate it have potential to augment current understanding of *T*. *equi* biology and further the development of effective control and preventive measures against equine piroplasmosis.

## Methods

### In vitro cultures of *T. equi*

*T. equi* (USDA strain) was maintained in vitro in an atmosphere 5% O_2_ and 5% CO_2_ at 37 °C, as previously described^38^. Briefly, *T*. *equi* was grown in purified horse red blood cells (RBCs) using M199 medium supplemented with 40% horse serum and hypoxanthine (Sigma-Aldrich, Tokyo, Japan) at 13.6 μg/ml. An antibiotic–antimycotic solution, containing 60 U/ml penicillin G, 60 μg/ml streptomycin, and 0.15 μg/ml amphotericin B (Sigma-Aldrich, Tokyo, Japan), was also added at a final concentration of 1% (v/v).

### Blasticidin susceptibility of *T. equi*

An in vitro growth inhibition assay was conducted to determine the half-maximal inhibitory concentration (IC_50_) of blasticidin against *T*. *equi*, as previously described^39^. Briefly, *T*. *equi* cultures at 10% hematocrit and 1% initial parasitaemia were treated with blasticidin at 0.8, 1.6, 3.1, 6.3, 12.5, 25, and 50 µM for 4 days. The medium, which contained 1% Milli-Q water that was equal to the volume of Milli-Q water used to dissolve the highest concentration of blasticidin, served as the non-treated control. The parasitaemias, which were determined 96 h after cultivation by counting the number of infected RBCs among > 5,000 RBCs in the Giemsa-stained RBC smears using a light microscope, were converted to growth rates relative to the non-treated control. IC_50_ values were calculated using GraphPad Prism (GraphPad Software, San Diego, CA, USA). These experiments were conducted in triplicate and repeated thrice.

### Sequencing ef-1α mRNA from *T. equi*

Total RNA was extracted from 200 μl of *T*. *equi*-infected RBCs using TRIzol reagent (Invitrogen, Carlsbad, CA, USA) according to the manufacturer’s instructions. RNA was purified using the QIAamp RNA Blood Mini Kit (Qiagen, Tokyo, Japan) with recombinant DNase I digestion (Takara, Otsu, Japan). This RNA was subjected to cDNA synthesis using the SMARTer RACE cDNA Amplification Kit (Takara, Kyoto, Japan). Forward (5′-GATTACGCCAAGCTTCGTGAGCATGCCTTGTTGGCCTTC -3′) and reverse primers (5′-GATTACGCCAAGCTTGGTCCTCCTTGTAGTCGCACTTG-3′), which were designed according to the mRNA sequences of the *ef-1α* genes from *T*. *equi* (WA strain, XM_004833157.1), *B. bovis* (AK441624.1), *B. bigemina* (KT439182.1), and *T*. *parva* (XM_761154.1), were used in 5′-RACE PCR and 3′-RACE PCR, respectively. The amplified PCR products were then cloned into a pRACE vector, transformed into *Escherichia coli* (Stellar Competent Cells) using the In-Fusion HD Cloning Kit (Takara, Kyoto, Japan), and then sequenced using the BigDye Terminator v3.1 Cycle Sequencing kit (Applied Biosystems, Tokyo, Japan). The sequences of the 5′-RACE and 3′-RACE PCR products were assembled and analysed using Basic Local Alignment Search Tool (BLAST; https://blast.ncbi.nlm.nih.gov/Blast.cgi) software and the EMBOSS needle pairwise sequence alignment tool (https://www.ebi.ac.uk/Tools/psa/emboss_needle/).

### Amplification and sequencing of long DNA fragments of elongation factor-1 alpha locus

gDNA was extracted from 200 µl of *T*. *equi-*infected RBCs using the QIAamp DNA Blood Mini Kit (Qiagen, Tokyo, Japan) with RNase A digestion (Takara, Otsu, Japan), according to the manufacturer’s instructions. The entire *ef-1α* gene locus (5.46-kbp) was amplified using a set of forward and reverse PCR primers designed against *rdrs* (5′-TCAGCCAAAATAGCGCACAAAAACTCCC-3′) and *gluRS* (5′-CCCAAAGCAACCCAAGCCATCTATG-3′) genes, respectively. KOD Fx Neo DNA polymerase, PCR buffer for KOD FX Neo and dNTPs (Toyobo, Osaka, Japan) were used according to the manufacturer’s instructions. The PCR amplicon was cloned into pCR 4Blunt-TOPO vector, transformed into TOP10 *E*. *coli* (Invitrogen, Carlsbad, CA, USA), and then sequenced using the BigDye Terminator v3.1 Cycle Sequencing kit (Applied Biosystems).

### Transfer plasmid construction

The homologous A and B flanking fragments and the promoter and terminator regions of the *ef-1α* gene were amplified from the 5.46-kbp fragment-containing pCR 4Blunt-TOPO vector using the PCR primers listed in Supplementary Table S2. The coding region of eGFP/BSD was also amplified from the pTracer-CMV/Bsd Mammalian Expression Vector (Invitrogen, Carlsbad, CA, USA). Homologous flanking fragments A and B were inserted into a Bluescript plasmid after digestion with *Sac*I and *Xma*I restriction enzymes (New England Biolabs, Ipswich, MA, USA), respectively, using the In-Fusion HD Cloning Kit (Takara, Kyoto, Japan), followed by insertion of the *Xba*I-digested terminator region of the *ef-1α* gene between the A and B fragments. In parallel, the *ef-1α* gene’s promoter sequence and the eGFP/BSD coding region were inserted into a second Bluescript plasmid after digestion with S*ac*I or *Xba*I, respectively. The fragment containing the promoter and the eGFP/BSD coding region was then amplified from the second plasmid, digested with *Nar*I, and inserted into the first plasmid between flanking fragment A and the terminator. The constructed plasmid containing the eGFP/BSD-expression cassette was purified using Qiagen Plasmid Maxi Kit (Qiagen, Tokyo, Japan), according to the manufacturer’s instructions.

### Parasite transfections

The eGFP/BSD-expression cassette-containing transfer plasmid was digested with *Apa*LI (New England Biolabs) before its transfection into *T*. *equi*. At 10% parasitaemia, the *T. equi*-infected RBCs were washed twice with phosphate-buffered saline. Linearised plasmids (20 μg) were resuspended in 100 µl of nucleofector human T-cell solution (Lonza, Cologne, Germany) and then mixed with pre-washed, 100-µl-infected RBCs. *T. equi* was then transfected with the linearised plasmid via a Nucleofector 2b device (Lonza) using the V-024 program. The transfectant was then immediately added to 1 ml of culture medium containing fresh RBCs at 10% hematocrit. At 24 h post-transfection, the culture containing transfected *T*. *equi* was treated daily with blasticidin at 70-µM (10 × IC_50_). Two weeks later, a single clonal line was isolated by limiting dilution. Infected RBCs (1 μl) from the clonal line were used for fluorescence microscopic observations (1,000 × magnification, Keyence, Pasadena, CA, USA). Parasite nuclei were stained with Hoechst 33,342 (Invitrogen, Carlsbad, CA, USA).

### Confirming genomic integration and expression of the eGFP/BSD

Genomic integration of eGFP/BSD-expression cassette was confirmed using PCR assays and Southern blot analyses. The PCR assays were conducted using gDNAs extracted from transgenic and parental lines with forward and reverse primers (Supplementary Table S1) targeting homologous flanking fragments A and B, eGFP/BSD and the *gluRS* gene, and eGFP/BSD and the *rdrs* gene, respectively. PCR assays using forward primers targeting flanking region A, promoter, eGFP/BSD coding region, terminator and flanking region B, together with the *gluRS*-targeting reverse primer were also conducted. The presence or absence of PCR amplicons and their sizes were compared between the transgenic and parental lines.

For the Southern blot analyses, gDNAs (2 µg) extracted from the transgenic and parental lines were digested with *Eco*RV restriction enzyme, electrophoresed on a 0.8% agarose gel, stained with Midori Green Advance DNA-staining (Nippon Genetics, Tokyo, Japan), and then transferred onto Hybond N^+^ (GE Health-care, Buckinghamshire, UK). Two DNA probes targeting the eGFP/BSD coding or promotor region were prepared using two sets of forward (5′-CCAAAGGAGAAGAACTTTTCACTGG-3′ or 5′-TTTGTGTCCGAGAATGTTTCC-3′, respectively) and reverse (5′-CCAAAGGAGAAGAACTTTTCACTGG-3′ or 5′-TTTGTGTCCGAGAATGTTTCC-3′, respectively) primers. The probes were labelled with thermostable alkaline phosphatase using the Alkphos Direct kit (GE Healthcare), according to the manufacturer’s instructions. Reactions between the labelled probes and DNA were detected using CDP-star detection reagent (GE Healthcare).

We also confirmed the expression of eGFP/BSD in transgenic *T*. *equi* in Western blot assay. Briefly, 180 µL of parasite-infected erythrocytes with ~ 5% to 8% parasitaemia were treated with 0.15% saponin, and then lysed in phosphate buffered-saline containing 1% Triton X-100 (Sigma-Aldrich, Tokyo, Japan) and protease inhibitor (Roche Diagnostics, Mannheim, Germany). The lysate was separated in 12.5% acrylamide gel, and then transferred onto a nitrocellulose membrane (Bio-Rad Laboratories, Feldkirchen, Germany). The membrane was blocked using 5% skim milk (Wako, Osaka, Japan), stained with anti-GFP rabbit polyclonal antibody at 1:500 (v/v) dilution (Thermo Fisher Scientific, MA, USA), and then incubated with horseradish peroxidase-conjugated anti-rabbit IgG donkey polyclonal antibody at 1:10,000 (v/v) dilution (GE Healthcare). The reaction was detected with Amersham ECL Western Blotting Detection Reagents (GE Healthcare) using ImageQuant LAS 500 chemiluminescence CCD camera system (GE Healthcare), according to the manufacturer’s instructions.

### In vitro growth rate assessments

The transgenic and parental lines (1% parasitaemia) were cultured in vitro using horse RBCs at 10% haematocrit. The cultures were treated with 70-µM blasticidin for 4 days. In addition, the transgenic and the parental lines were grown in normal medium without blasticidin. Parasitaemia was microscopically monitored using Giemsa-stained RBC smears prepared every 24 h until 96 h post-cultivation. This experiment was conducted in triplicate and repeated three times.

### Ethics approval

All experiments were approved by the Animal Care and Use Committee and the Biological Safety Committee of Obihiro University of Agriculture and Veterinary Medicine, Hokkaido, Japan (Approval numbers: 18-118) and were conducted according to the Fundamental Guidelines for Proper Conduct of Animal Experiments and Related Activities in Academic Research Institutions under the jurisdiction of the Ministry of Education, Culture, Sports, Science and Technology of Japan.

## Supplementary Information

Supplementary Information.

## Data Availability

Any requests for materials should be addressed to Naoaki Yokoyama (DVM, PhD); E-mail: yokoyama@obihiro.ac.jp.
