# Supplementary material for: Development of a stable transgenic Theileria equi parasite expressing an enhanced green fluorescent protein/blasticidin S deaminase
Source: Sci Rep. 2021 Apr 27;11:9107. doi: 10.1038/s41598-021-88594-w (PMC8079379; doi:10.1038/s41598-021-88594-w)
Supplement: Supplementary file 1 — Supplementary Information. [file 41598_2021_88594_MOESM1_ESM.pdf]

Supplementary Information

Development of a stable transgenic *Theileria equi* parasite expressing an enhanced green fluorescent protein/blasticidin S deaminase

Bumduuren Tuvshintulga[1,2†], Arifin Budiman Nugraha[1,3†], Tomoka Mizutani[1], Mingming Liu[1], Takahiro Ishizaki[4], Thillaiampalam Sivakumar[1], Xuenan Xuan[1], Naoaki Yokoyama[1*], & Ikuo Igarashi[1]

[1]National Research Center for Protozoan Diseases, Obihiro University of Agriculture Veterinary Medicine, Inada-cho, Obihiro, Hokkaido 080-8555, Japan.
[2]Institute of Veterinary Medicine, Mongolian University of Life Sciences, Zaisan 17024, Ulaanbaatar, Mongolia.
[3]Department of Animal Infectious Diseases and Veterinary Public Health, Faculty of Veterinary Medicine, IPB University, Jl. Agatis, Kampus IPB Dramaga, Bogor, Jawa Barat 16680, Indonesia.
[4]Department of Protozoology, Institute of Tropical Medicine (NEKKEN), Nagasaki University, Nagasaki 852-8523, Japan.

[†]B. Tuvshintulga and A.B. Nugraha equally contributed this work.

*Correspondence note: Naoaki Yokoyama (DVM, PhD); E-mail: yokoyama@obihiro.ac.jp

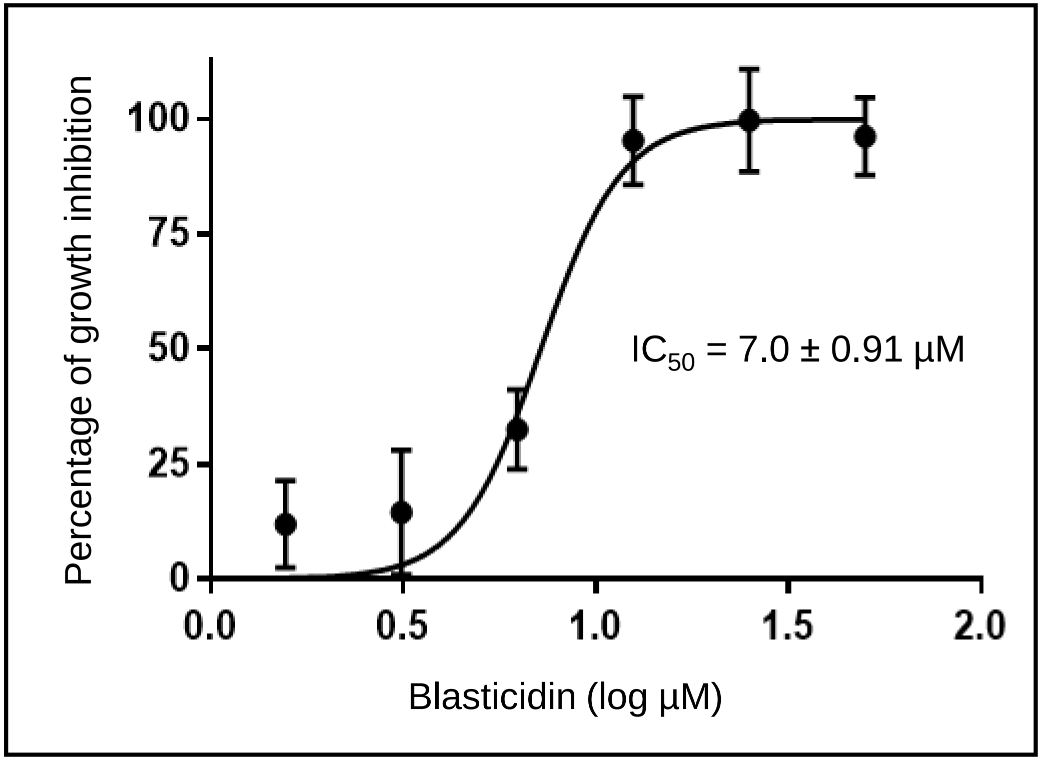

Supplementary Figure S1. Blasticidin sensitivity in *T. equi*. *In vitro* cultures of *T. equi* were treated with blasticidin at various concentrations for 4 days. The percentage of growth inhibition was calculated from the parasitaemia reduction in treated cultures compared with the non-treated control. They were plotted against the logarithm for the drug concentrations, and the half maximal inhibitory concentration (IC_{50}) was calculated. Mean standard error values were calculated from three independent experiments.

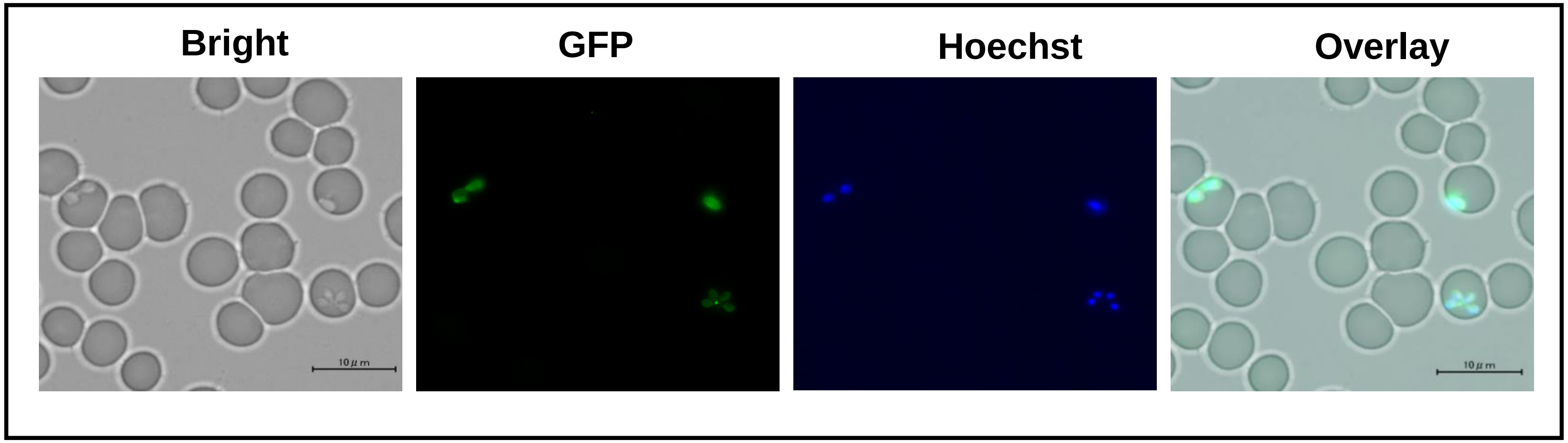

Supplementary Figure S2. Visualisation of *T*. *equi* on day 12 post-transfection. Ring, paired, and Maltese cross forms of the transgenic parasites emit a green fluorescent signal. Parasite DNA was stained with Hoechst 33342.

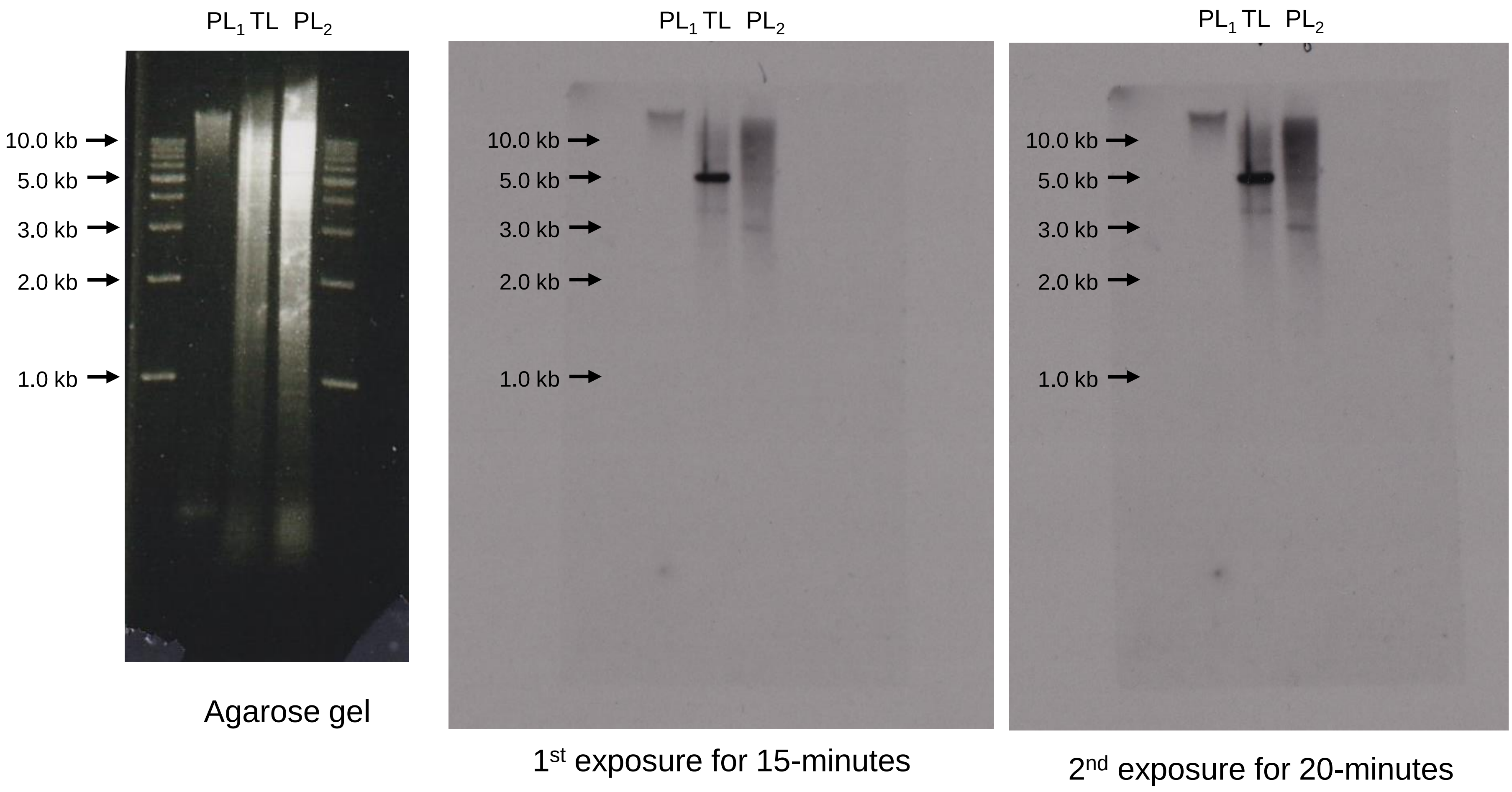

Supplementary Figure S3. Agarose gel and X-ray film images of Southern blot analysis. Genomic DNA extracted from the transgenic *T*. *equi* line (TL) and parental line (PL_2) was subjected to Southern blotting analysis after digestion with *Eco*RV using a probe targeting *green fluorescent protein* gene to confirm that the enhanced green fluorescent protein/blasticidin S deaminase-expression cassette was integrated into a single genomic locus. (see Fig. 5a, c and d). Undigested genomic DNA of parental line (PL_1) was used as a control.

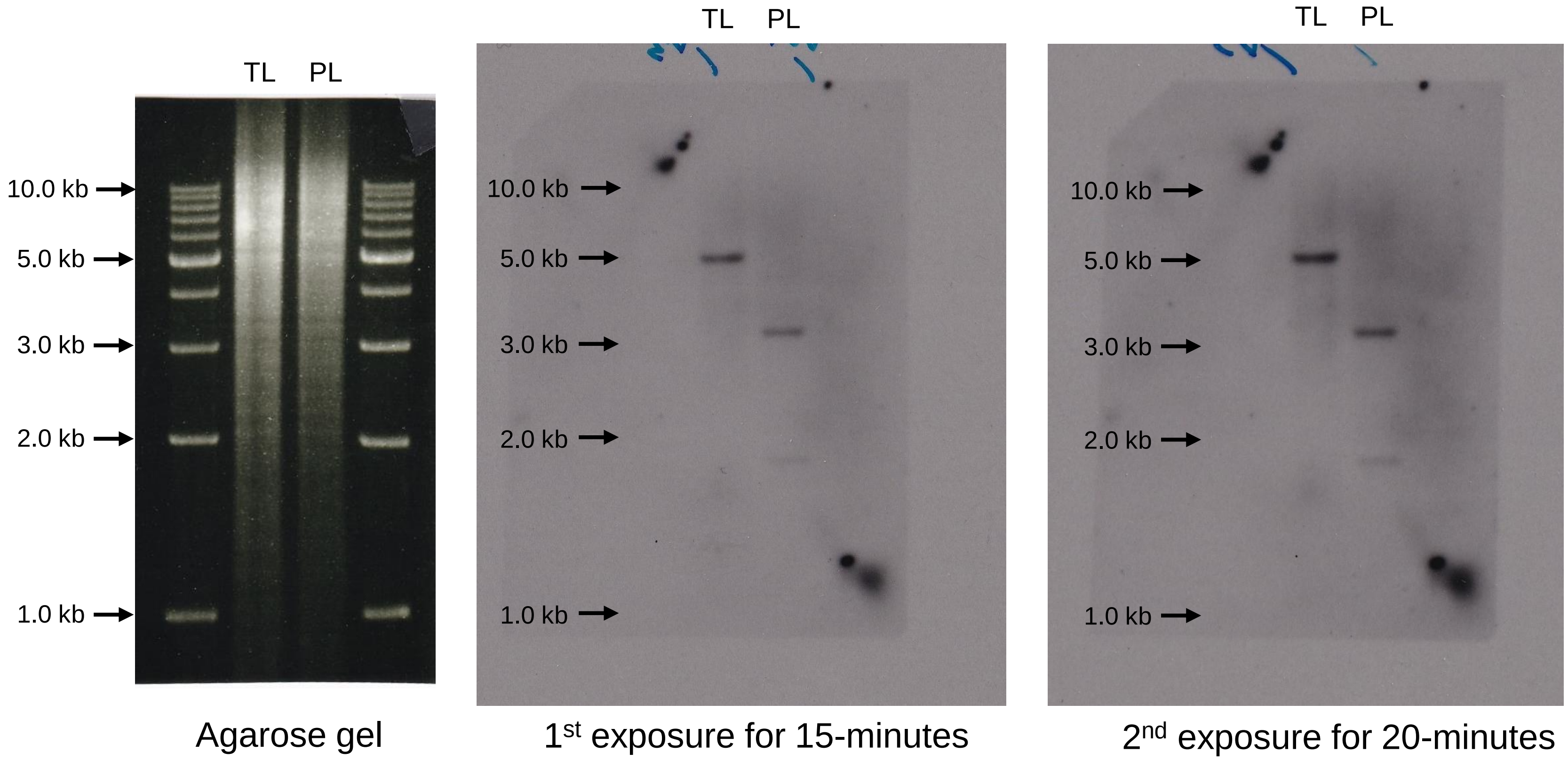

Supplementary Figure S4. Agarose gel and X-ray film images of Southern blot analysis. Genomic DNA extracted from the transgenic *T*. *equi* line (TL) and parental line (PL_2) was subjected to Southern blotting analysis after digestion with *Eco*RV using a probe targeting promoter region of the *elongation factor-1 alpha* gene to confirm that the enhanced green fluorescent protein/blasticidin S deaminase-expression cassette was integrated into a single genomic locus. (see Fig. 5b, c and d).

Supplementary Table S1. Primers used for the confirmation of genomic integration of eGFP/BSD-expression cassette in transgenic *T*. *equi*.

Targets	PCRs	Primers	Oligonucleotide sequences (5′ to 3′)	Ta
Flank A of *ef-1α*	PCR1	1	AACATGATTACTGGTACCTCTCAGGCTGATGTTGC	72℃
Flank B of *ef-1α*		2	GGGACAGTACCAATACCACCAATCTTGTATACACC	
Rdrs	PCR2	3	TCAGCCAAAATAGCGCACAAAAACTCCC	66℃
Flank A of *ef-1α*		1	AACATGATTACTGGTACCTCTCAGGCTGATGTTGC	
Gfp	PCR3	4	AAGTTTCCGATAAAGGCGCCATGGCCTCCAAAGGAGAAG	68℃
GluRS		5	CCCAAAGCAACCCAAGCCATCTATG	
Flank A of *ef-1α*	PCR4	1	AACATGATTACTGGTACCTCTCAGGCTGATGTTGC	68℃
GluRS		5	CCCAAAGCAACCCAAGCCATCTATG	
IG of *ef-1α*	PCR5	6	ACAAGATGCCATGGTACAGGCGCCTGCATCATAATCCTGACG	68℃
GluRS		5	CCCAAAGCAACCCAAGCCATCTATG	
Gfp	PCR6	4	AAGTTTCCGATAAAGGCGCCATGGCCTCCAAAGGAGAAG	68℃
GluRS		5	CCCAAAGCAACCCAAGCCATCTATG	
Ter of *ef-1α*	PCR7	7	TGTGGGAGGGCTAATCTAGAAATCAACTTTTATATTCAACG	68℃
GluRS		5	CCCAAAGCAACCCAAGCCATCTATG	
Flank B of *ef-1α*	PCR8	8	TTATTATATTGATATCTAGAAGGGCAAGATTTTGGTCGAG	66℃
GluRS		5	CCCAAAGCAACCCAAGCCATCTATG	

ef-1α, *elongation factor-1 alpha*; *gfp*, *green fluorescent protein*; *bsd*, *blasticidin S deaminase*; *rdrs*, *ribonucleoside-diphosphate reductase*; *gluRS*, *glutamyl-tRNA synthetase*; IG, intergenic region; Ter, terminator; Ta, optimal annealing temperature.

Supplementary Table S2. Primers used for the amplification of the components of eGFP/BSD-expression cassette.

Targets	Primers	Oligonucleotide sequences (5′ to 3′)
Homologous flanking region A of *ef-1α*	F	AATTG**GAGCTC**ATGGGTAAGGAAAAGACTC
Homologous flanking region A of *ef-1α*	R	**GGCGCC**TGTACCATGGCATCTTGT
IG of *ef-1α*	F	ACAAGATGCCATGGTACA**GGCGCC**TGCATCATAATCCTGACG
IG of *ef-1α*	R	**GGCGCC**TTTATCGGAAAACTTGCT
gfp	F	TTTCCGATAAA**GGCGCC**ATGGCCTCCAAAGGAGAAG
bsd	R	ATATAAAAGTTGATT**TCTAGA**TTAGCCCTCCCACACATA
Ter of *ef-1α*	F	GATGCCATGGTACA**GGCGCC**TCTAGAAATCAACTTTTATATTCAACG
Ter of *ef-1α*	R	CCAAAATCTTGCCCT**TCTAGA**TATCAATATAATAAATAG
Homologous flanking region B of *ef-1α*	F	TTATTATATTGATA**TCTAGA**AGGGCAAGATTTTGGTCGAG
Homologous flanking region B of *ef-1α*	R	TCGAATTCCTGCAG**CCCGGG**TTACTTCTTGGCAGCCTTA

Ef-1α, *elongation factor-1 alpha*; IG, intergenic region; *gfp*, *green fluorescent protein*; *bsd*, *blasticidin S deaminase*; Ter, terminator; recognition sites of restriction endonucleases are highlighted in bold; overlapping regions for fusion of DNA fragments into plasmid are underlined.
